# Global Warming Threshold and Mechanisms for Accelerated Greenland Ice Sheet Surface Mass Loss

**DOI:** 10.1029/2019MS002029

**Published:** 2020-09-09

**Authors:** Raymond Sellevold, Miren Vizcaíno

**Affiliations:** ^1^ Geoscience and Remote Sensing Delft University of Technology Delft the Netherlands

## Abstract

The Community Earth System Model version 2.1 (CESM2.1) is used to investigate the evolution of the Greenland ice sheet (GrIS) surface mass balance (SMB) under an idealized CO_2_ forcing scenario of 1% increase until stabilization at 4× pre‐industrial at model year 140. In this simulation, the SMB calculation is coupled with the atmospheric model, using a physically based surface energy balance scheme for melt, explicit calculation of snow albedo, and a realistic treatment of polar snow and firn compaction. By the end of the simulation (years 131–150), the SMB decreases with 994 Gt yr^−1^ with respect to the pre‐industrial SMB, which represents a sea‐level rise contribution of 2.8 mm yr^−1^. For a threshold of 2.7‐K global temperature increase with respect to pre‐industrial, the rate of expansion of the ablation area increases, the mass loss accelerates due to loss of refreezing capacity and accelerated melt, and the SMB becomes negative 6 years later. Before acceleration, longwave radiation is the most important contributor to increasing energy for melt. After acceleration, the large expansion of the ablation area strongly reduces surface albedo. This and much increased turbulent heat fluxes as the GrIS‐integrated summer surface temperature approaches melt point become the major sources of energy for melt.

## Key Points

- GrIS surface mass loss accelerates for a global warming of 2.7 K through increased surface melt and loss of refreezing capacity
- Longwave radiation is the main contributor to melt increase before acceleration; albedo and turbulent fluxes add major contributions after
- Anthropogenic‐forced atmospheric circulation changes (North Atlantic Oscillation and Greenland blocking index) partially reduce melt

## 1 Introduction

Since the 1990s, the Greenland ice sheet (GrIS) has lost mass (Bamber et al., 2018; King et al., 2018; Shepherd et al., 2012). This mass loss has further accelerated since around 2000 (Bamber et al., 2018). The cumulative mass loss from Greenland since the 2000s is equivalent to ∼11 mm of sea‐level rise (Bamber et al., 2018). Both increasing ice discharge and a decreased surface mass balance (SMB) contribute to the mass loss. Of these, the SMB is the dominant contributor (Fettweis et al., 2017; van den Broeke et al., 2016), and the acceleration in mass loss is attributed to accelerated SMB decline (Enderlin et al., 2014). Proposed driving mechanisms behind the accelerated surface mass loss are changes in North Atlantic circulation (Delhasse et al., 2018; Fettweis, Hanna, et al., 2013; Hanna et al., 2018), albedo‐melt feedback (Box et al., 2012), depletion of firn refreezing capacity (Vandecrux et al., 2019), and the hypsometric geometry of the GrIS (van As et al., 2017).

State‐of‐the‐art modeling project reductions of GrIS SMB. These projections are made with either simple positive degree day calculations (Golledge et al., 2019; Yoshimori & Abe‐Ouchi, 2012), using regional climate models (RCMs) (Fettweis, Franco, et al., 2013; Franco et al., 2013; Mottram et al., 2017; Rae et al., 2012; van Angelen et al., 2013) or earth system models (Vizcano et al., 2014). There is a consensus among these studies that future SMB decline is due to increased surface melt and runoff, with a small offset due to increased snow accumulation in the interior. However, the magnitude of the SMB decline varies greatly. Scenario uncertainty and model sensitivity to CO_2_ are the greatest contributors to this uncertainty (Fettweis, Franco, et al., 2013).

Here we present projections of GrIS SMB with the Community Earth System Model version 2.1 (CESM2.1) under an idealized high CO_2_ scenario. CESM2.1 produces realistic present‐day GrIS SMB, both through its interactive calculation (van Kampenhout et al., 2020) and as a driving model of RCM downscaling (Noël et al., 2020). The SMB is calculated in the land component and is downscaled through elevation classes (Sellevold et al., 2019), with a prognostic albedo, and an advanced snow‐model fit for applications to polar ice sheets (van Kampenhout et al., 2017). This paper seeks to answer the following scientific questions: What is the modeled SMB evolution in response to CO_2_? What are the mechanisms involved in the surface mass change? What is the impact of future changes in atmospheric circulation on the SMB?

The model, experimental setup, and analysis methods are described in section 2. Section 3 shows an overview of projected global and Arctic changes. GrIS SMB projections and processes are described in section 4, with linkages to atmospheric circulation metrics in section 5. We make a summary, discussion, and conclusions in section 6.

## 2 Method

### 2.1 Model

The model used for this study is CESM2.1 (Danabasoglu et al., 2020). This model features a fully coupled atmosphere, ocean, sea ice, land, and ice sheet components. The atmospheric model is the Community Atmosphere Model version 6. This model uses a finite‐volume dynamical core at 0.9° (latitude) × 1.25° (longitude) horizontal resolution, with 32 vertical levels where the model top is at 3.6 hPa. This model features a new subgrid orographic drag parameterization (Beljaars et al., 2004), new cloud microphysics (Gettelman & Morrison, 2015), and a new subgrid cloud parameterization (Bogenschutz & Krueger, 2013). The ocean component is the Parallel Ocean Program version 2 (Danabasoglu et al., 2012; Smith et al., 2010) with a nominal resolution of 1°. The ocean model uses 60 vertical levels, with a maximum depth of 5,500 m. The sea ice is simulated with the Los Alamos Sea Ice model version 5 (Hunke et al., 2017) at the same grid as the ocean model.

The land model is the Community Land Model version 5 (Lawrence et al., 2019). This model now features a realistic representation of polar snow (van Kampenhout et al., 2017), which allows for an explicit and realistic calculation of snow refreezing and extending the snow cap from 1‐m water equivalent in the previous generation CESM model to 10‐m water equivalent. The simulation of melt over glaciated surfaces is done through the use of elevation classes to account for subgrid topographical variations (Sellevold et al., 2019). For each of the 10 elevation classes, the near‐surface atmospheric temperature is downscaled using a fixed lapse rate of 6 K km^−1^, the near‐surface humidity is downscaled by assuming fixed relative humidity, and the incoming longwave radiation is downscaled with a fixed lapse rate of 32 W m^−2^ km^−1^. The phase of precipitation is also downscaled to the elevation classes based on near‐surface air temperature. At temperatures lower than −2°C, precipitation falls purely as snow; at temperatures higher than 0°C, precipitation falls exclusively as rain.

The melt is calculated at each elevation class independently with a surface energy balance (SEB) scheme. The scheme computes melt energy *M* (in W m^−2^) from the sum of radiative, turbulent, and conductive fluxes at the ice sheet surface:

(1) $$M={\text{SW}}_{net}+{\text{LW}}_{net}+\text{SHF}+\text{LHF}+\text{GHF}$$

where SW_*net*_ is net shortwave radiation, LW_*net*_ is net longwave radiation, SHF is the sensible heat flux, LHF is the latent heat flux, and GHF is the ground heat flux.

This equation can be rewritten more specifically as

(2) $$M={\text{SW}}_{in}(1-\alpha )+{\text{LW}}_{in}-\sigma_{sb}\epsilon T_{sfc}^{4}+\text{SHF}+\text{LHF}+\text{GHF}$$

where SW_*in*_ is the incoming shortwave (solar) radiation, *α* is the albedo, LW_*in*_ is the incoming longwave radiation, *σ*_*sb*_ is the Stefan Boltzman constant, *ϵ* is the surface emissivity, and *T*_*sfc*_ is the surface temperature. For bare ice, the albedo is fixed to 0.5 in the visible spectrum and 0.3 in the near‐infrared spectrum. The snow albedo is prognostically simulated (Flanner & Zender, 2006).

The SMB (in mm of water equivalent) is calculated at each elevation class as

(3) SMB=SNOW+REFREEZING−MELT−SUBLIMATION

The SMB, and its components, is then represented by the area‐weighted average across the elevation classes at the lower resolution (same as atmospheric component) grid cell of the land model.

The land ice model is the Community Ice Sheet Model (CISM) version 2.1 (Lipscomb et al., 2019). This model has a default horizontal resolution of 4 km for the Greenland domain. In this study, ice sheet evolution is turned off, so CISM2.1 is used purely as a diagnostic model to output downscaled SMB. The SMB downscaling from elevation classes to CISM is done through a bilinear horizontal interpolation and vertical linear interpolation. SMB in CISM does not account for snowmass variations, as in the land model, but only variations in ice mass.

### 2.2 Simulations

The control simulation (CTRL) is a pre‐industrial simulation with a fixed atmospheric CO_2_ concentration of 284.7 ppm (Danabasoglu et al., 2019). This simulation participates in the Tier 1 simulations of the Coupled Model Intercomparison Project (CMIP) 6 Diagnostic, Evaluation and Characterization of Klima (DECK) experiments (Eyring et al., 2016). It is ∼1,200 years long. Here we only use the years 501–650 from the CTRL simulation, as our sensitivity simulation is branched off at year 501 and run for 150 years. As the pre‐industrial simulation is at steady state, 150 years are sufficient to sample the unforced climate variability.

To assess the response of the GrIS SMB to CO_2_ forcing, we use a 1% increase in CO_2_ concentration per year, until 4× pre‐industrial CO_2_ concentration (1PCT Danabasoglu, 2019). After reaching stabilization at 1,140 ppm of CO_2_ concentration, the CO_2_ forcing is kept constant. The simulation is 150 years long. This simulation is also participating as a Tier 1 CMIP6 DECK simulation.

A longer simulation with the same greenhouse gas forcing and a dynamical GrIS is analyzed in Muntjewerf et al. (2020). In the current study, the main focus is on the coupling between the atmosphere and the GrIS SMB, with detailed analysis of SMB and SEB components.

### 2.3 Analysis

#### 2.3.1 Oceanic and Atmospheric Circulation Metrics

The North Atlantic Meridional Overturning Circulation (NAMOC) index is calculated from annual values as the maximum of the overturning stream function north of 28°N to 90°N and below 500‐m depth.

The North Atlantic Oscillation (NAO) is calculated as the leading empirical orthogonal function (EOF) of the seasonal mean (December–February: DJF, and June–August: JJA) sea‐level pressure in the North Atlantic region (20°N to 80°N, and 90°W to 40°E (Hurrell, 1995; Hurrell & Deser, 2010). The NAO index is calculated from the resulting principal component (PC) time series and standardized with respect to the index from CTRL.

To calculate the Greenland blocking index (GBI), we use the revised index from Hanna et al. (2018). The procedure to calculate this index is as follows:

1. We make seasonal means (DJF and JJA) of 500‐hPa geopotential heights (Z_500_). The next steps are applied to the seasonal averages independently.
2. Calculate the area‐averaged Z_500_ over the Greenland region (60°N to 80°N, and 80°W to 20°W).
3. Calculate the area‐averaged Z_500_ over the Arctic region (60°N to 80°N).
4. Subtract the Arctic averaged Z_500_ from the Greenland Z_500_.
5. The resulting time series is standardized with respect to the CTRL.

The North Atlantic jet latitude is calculated with the formula from Woollings et al. (2010). The calculation is as follows:

1. Daily zonal winds at 700, 775, 850, and 925 hPa are vertically averaged.
2. We extract data in the region 15°N to 75°N and 60°W to 0°W based on the resulting profile from (1).
3. The resulting profile is zonally averaged.
4. We then apply a Lanczos low‐pass filter with 61 weights and a 10‐day cutoff frequency to remove winds associated with individual synoptic systems.
5. The jet latitude is the latitude where we find the maximum zonal wind speed.

The NAO, GBI, and jet latitude are further decomposed into a sub‐decadal and decadal component. To extract sub‐decadal variations, we use a Lanczos high‐pass filter with 21 weights and a cutoff frequency of 10 years. For the decadal component, we use a 10‐ to 30‐year Lanczos band‐pass filter with 21 weights.

#### 2.3.2 Composite and Trend Analysis

For maps illustrating responses to greenhouse gas forcing, we compare the last 20 years of the 1PCT simulation with the entire CTRL simulation. We use a Wilcoxon *t*‐test with a threshold of *p*<0.01 to test the significance of responses. The choice of using the Wilcoxon *t*‐test instead of the more common Student's *t*‐test is our expectation of a change in variability and the different sample sizes of our CTRL and the last 20 years of the 1PCT simulation.

To assess trends, we use linear least squares regression fits and consider trends as significant when *p*<0.01. Wherever the trends are nonlinear due to, for example, acceleration, we use piecewise linear regression fits and report on the slope and length of each of these.

To address the question of whether a CO_2_ forced signal has emerged or not from internal variability, we apply a similar metric as outlined by Fyke et al. (2014). We consider a signal emerged, if the 20‐year backward running mean is lower or higher than the mean ±2 standard deviations of the corresponding quantity from the CTRL. Also, we apply the condition that the running mean needs to stay lower or higher than this threshold for the rest of the simulation.

## 3 Global and Arctic Climate Change

**Figure 1:**
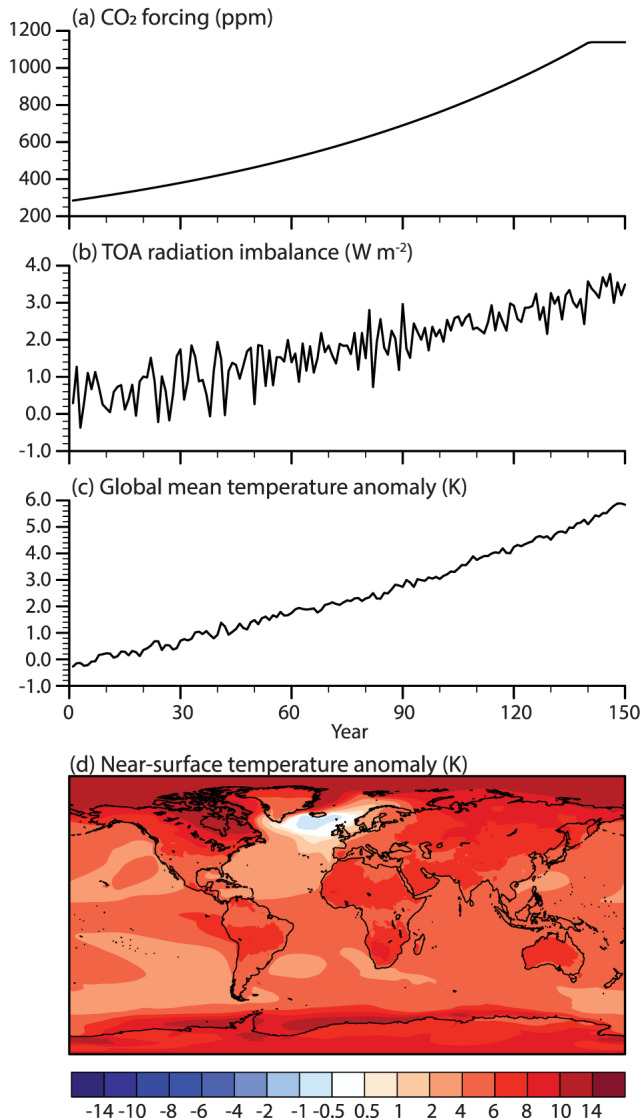
Global responses in the 1PCT simulation. Annual average time series of (a) CO_2_ forcing (ppm),(b) cumulative top‐of‐the‐atmosphere radiation imbalance (W m^−2^), (c) global mean temperature anomaly (K), and (d) map of change in global temperatures (K) for years 131–150 of 1PCT with respect to CTRL. Note the nonlinear color scale.

The response to the increased CO_2_ forcing (Figure 1a) is an increase in the amount of radiation in the earth system (Figure 1b). This leads to a rise in global mean surface air temperatures (*T*_2*m*_; Figures 1c and 1d). The radiation imbalance, defined as LW_*net*_ + SW_*net*_ at the top of the atmosphere, is increasing with time. In the last 20 years of the simulation, the imbalance is 3.2 ± 0.3 W m^−2^. Part of this excess energy increase is used to raise atmospheric temperatures. The global mean near‐surface temperature trend in the simulation is 0.04 K yr^−1^. The annual global mean temperature increase (Δ*T*_*global*_) by the end of the simulation (years 131–150) compared to CTRL is 5.3 ± 0.4 K. The Arctic region (north of 60°N) warms the most (Figure 1d), by 8.7 ± 1.0 K, or 1.6 times the global mean. Within the Arctic, the highest warming occurs over the ocean. Northern Canada, the Weddell Sea, and the Bellingshausen Sea are areas with high warming. The North Atlantic stands out, as it is the only region with cooling (of up to −1 K), in connection with a large slowdown in the NAMOC (supporting information Figure S1) (Bryden et al., 2020; Drijfhout et al., 2012). This NAMOC slowdown is a common feature in CESM2.1 (Muntjewerf et al., 2020).

**Figure 2:**
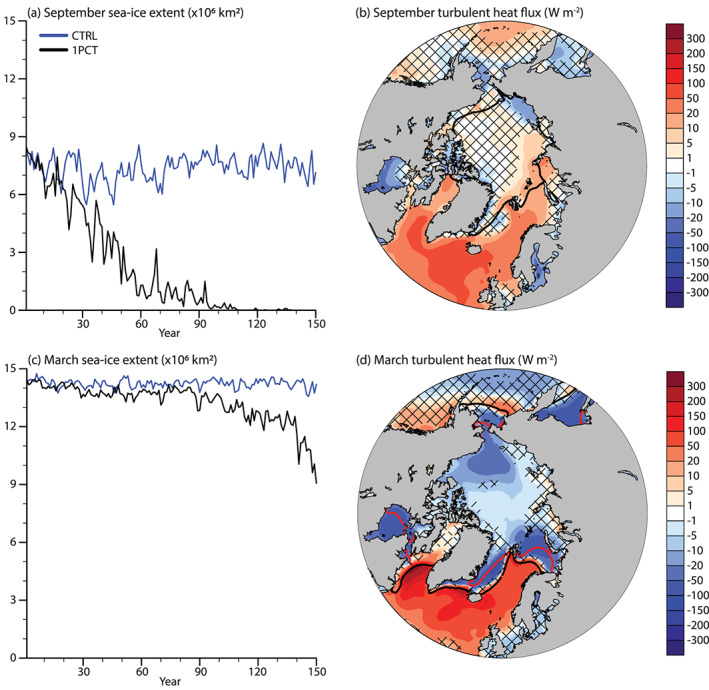
Sea ice responses to the CO_2_ forcing for (top) September and (bottom) March. (a, c) Sea ice extent (×10^6^ km^2^) and (b, d) turbulent heat flux anomaly (W m^−2^) for the years 131–150 compared to CTRL. Note the nonlinear color scales. For the time series (a, c) the blue line shows the CTRL, while the black line shows the 1PCT simulation. The sea ice extent is defined as the area north of 60°N where sea ice concentration is greater than 15%. For (b) and (d) the black line shows the sea ice extent from CTRL, and the red line shows the sea ice extent from the 1PCT. Positive turbulent heat flux means energy gain at the surface. Areas with non‐significant changes are patched.

A CO_2_ forced signal in September sea ice decline emerges by year 31, for a Δ*T*_*global*_ = 0.8 K (Figure 2a). Further, the Arctic becomes seasonally ice free (<1 × 10^6^ km^2^ sea ice extent) in year 72 at Δ*T*_*global*_ = 2.1 K. Despite this large reduction, the turbulent heat fluxes from the now sea ice‐free ocean do not significantly change (Figure 2b). Rather, the temperature and humidity increase of the atmosphere inhibits the turbulent transfer of heat and moisture from the ocean to the atmosphere over the Arctic ocean in September. In the North Atlantic, less turbulent heat is transferred from the ocean to the atmosphere. The largest reduction is collocated with the region of cooling in the North Atlantic (Figure 1d).

The yearly maximum (March) sea ice extent in the Arctic decreases with −2.9 ± 1.1 × 10^6^ km^2^ by years 131–150 with respect to CTRL. The anthropogenic signal can be separated from natural variability in year 89, when Δ*T*_*global*_ = 2.8 K. The ice edge retreats everywhere except for the Baffin Bay. Outside of the CTRL ice edge, the turbulent heat fluxes increase (i.e., there is less surface‐to‐atmosphere energy transfer) as in September, but the response is stronger. The strongest positive responses are located close to the GrIS. On the other hand, the turbulent heat fluxes decrease (i.e., more surface‐to‐atmosphere transfer) everywhere inside of the CTRL ice edge, due to reduced pan‐Arctic sea ice and snow thickness. Strongest responses are co‐located with the 1PCT ice edge and the Beaufort Gyre. The areas co‐located with the 1PCT ice edge experience a large decrease as the surface becomes ice free.

**Figure 3:**
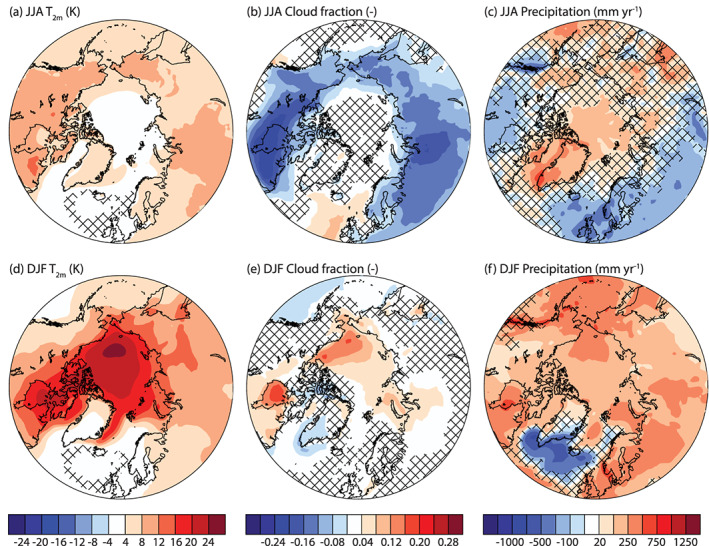
Overview of Arctic climate change in the last 20 years of the 1PCT simulation compared to CTRL for JJA (top) and DJF (bottom). (a, d) Near‐surface temperature (K), (b, e) cloud fraction (‐), and (c, f) precipitation (mm yr^−1^). Areas with non‐significant changes are patched. Note the nonlinear color scale for (c) and (f).

Increases in Arctic summer temperature by the end of 1PCT are strongest over land (Figure 3a), in connection with large snow cover decrease. Additionally, the cloud cover over the Arctic land is reduced (Figure  3b), increasing incoming solar radiation at the surface. The Arctic ocean warms less, likely due to the additional energy being used to melt sea ice and raise ocean temperatures. Summer precipitation increases over the Arctic, including the GrIS, and decreases over land toward midlatitudes (Figure 3c).

In winter, the warming over the Arctic ocean is strongest (Figure 3d), in connection with sea ice reduction and increased turbulent fluxes as already shown. The strongest local warming is over the Beaufort Gyre. This area also sees the strongest increase in cloud cover during winter (Figure 3e). As clouds increase the incoming longwave radiation, this contributes to generating the strongest warming here. Winter precipitation decreases along the southern Greenland margin and in the Greenland sea (Figure 3f). This precipitation decrease is co‐located with the lowering of near‐surface temperatures. It is likely that the decreased ocean‐to‐atmosphere fluxes of heat and moisture act to stabilize the atmosphere, resulting in fewer or weaker storms (Figure S2) and less precipitation here.

## 4 GrIS SMB and SEB Evolution

### 4.1 SMB Evolution

**Figure 4:**
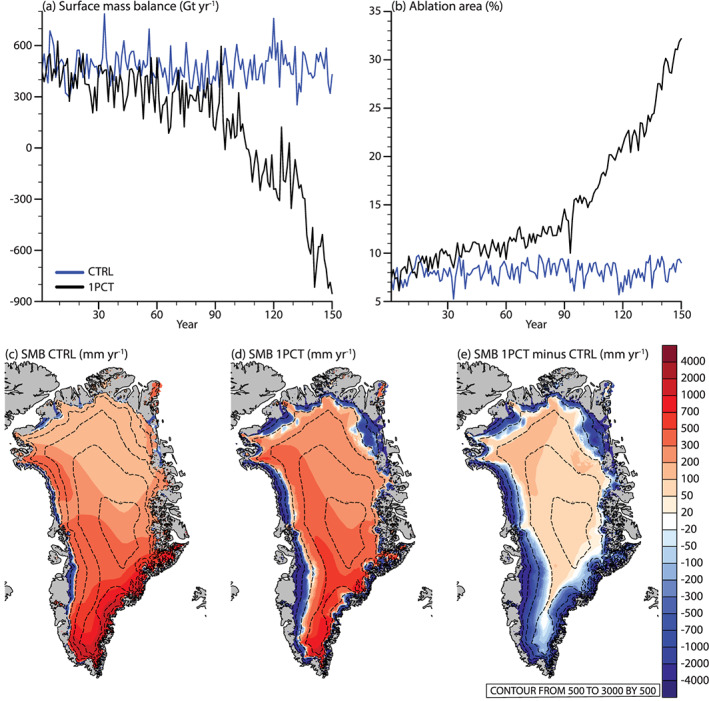
Annual ice sheet integrated (a) SMB (Gt yr^−1^) and (b) ablation area (%; as a percentage of total ice sheet area) for CTRL (blue) and 1PCT (black). Maps of annually averaged SMB (mm yr^−1^) at the ice sheet model grid (4 km) in (a) CTRL, (b) years 131–150 of 1PCT, and (c) SMB anomalies of years 131–150 of 1PCT with respect to CTRL. Note the nonlinear color scale. Dashed lines show surface elevation contours, starting with 500 up to 3,000 m by 500 m.

The SMB of the GrIS decreases with ∼994 Gt yr^−1^ in the 1PCT simulation (Figure 4a and Table 1). According to the criteria in section 2.3.2, we consider the CO_2_ forced SMB signal emerged from variability in year 90 (Δ*T*_*global*_ = 2.7 K). The SMB becomes negative in year 96 (Δ*T*_*global*_ = 3.0 K). In the first 90 years of the simulation, the trend is −2.5 ± 0.4 Gt yr^−2^. Around year 90, this trend transitions to −15.9 ± 1.1 Gt yr^−2^, which represent a sixfold increase.

**Table 1:** SMB and SMB Components in the CTRL, the Last 20 Years of 1PCT, and Their Difference (Diff)

Component	CTRL (Gt yr^−1^)	1PCT (Gt yr^−1^)	Diff (Gt yr^−1^)	Trend 1 (Gt yr^−2^)	Trend 2 (Gt yr^−2^)
SMB (4 km)	472 ± 91	−522 ± 239	− **994**	− **2.5** ± **0.4**	− **15.9** ± **1.1**
SMB	464 ± 99	−615 ± 285	− **1,081**	− **2.6** ± **0.5**	− **17.1** ± **1.4**
Snowfall	763 ± 75	757 ± 78	−6	0.3 ± 0.3	−0.1 ± 0.6
Rainfall	77 ± 14	257 ± 33	**180**	**0.6** ± **0.1**	**2.5** ± **0.2**
Refreezing	227 ± 36	692 ± 83	**466**	**2.0** ± **0.2**	**4.8** ± **0.5**
Melt	496 ± 81	2,036 ± 317	**1,540**	**5.9** ± **0.5**	**22.0** ± **1.5**
Sublimation	30 ± 2	30 ± 7	−1	**0.1** ± **0.0**	− **0.2 ** ± ** 0.0**

*Note*. Trend 1 is the linear regression slope between years 1 and 89; trend 2 is the slope in years 90–150. Differences and trends in bold are significant. SMB (4 km) only accounts for ice mass variations, while SMB also includes snowmass variations. The ± indicates the 1 standard deviation.

The ablation area expands from 8.1% (pre‐industrial) to 27.6% (years 131–150) during the 1PCT (Figure 4b). The CO_2_ forced signal emerges already in year 44 (Δ*T*_*global*_ = 1.0 K), 46 years before the anthropogenic SMB signal emerges. This is due to much lower interannual variability in ablation area.

The lower row of Figure 4 shows the SMB as simulated by CTRL, 1PCT, and their difference. The CTRL simulation (Figure 4c) shows SMB patterns comparable to present‐day SMB (Fettweis et al., 2017; Noël et al., 2016). There are two local accumulation maxima, one located in the South‐East and one in the North‐West. Ablation areas are along the margins, in western and northern parts of the ice sheet. The regionally heterogenous equilibrium line altitude is in the range 500–1,500 m.

The most striking SMB feature of the last 20 years of the 1PCT is the large expansion of the ablation areas (Figure 4d). This raises the equilibrium line with ∼500 m. The high accumulation area in the South‐East remains the area with the highest accumulation.

The anomaly map (Figure 4e) reveals that SMB is decreased along the margin, and up to approximately 2,000 m. On the other hand, SMB increases in the interior. This result is in line with 21st‐century projections of GrIS SMB (Mottram et al., 2017; Vizcano et al., 2014).

### 4.2 SMB Components Evolution

**Figure 5:**
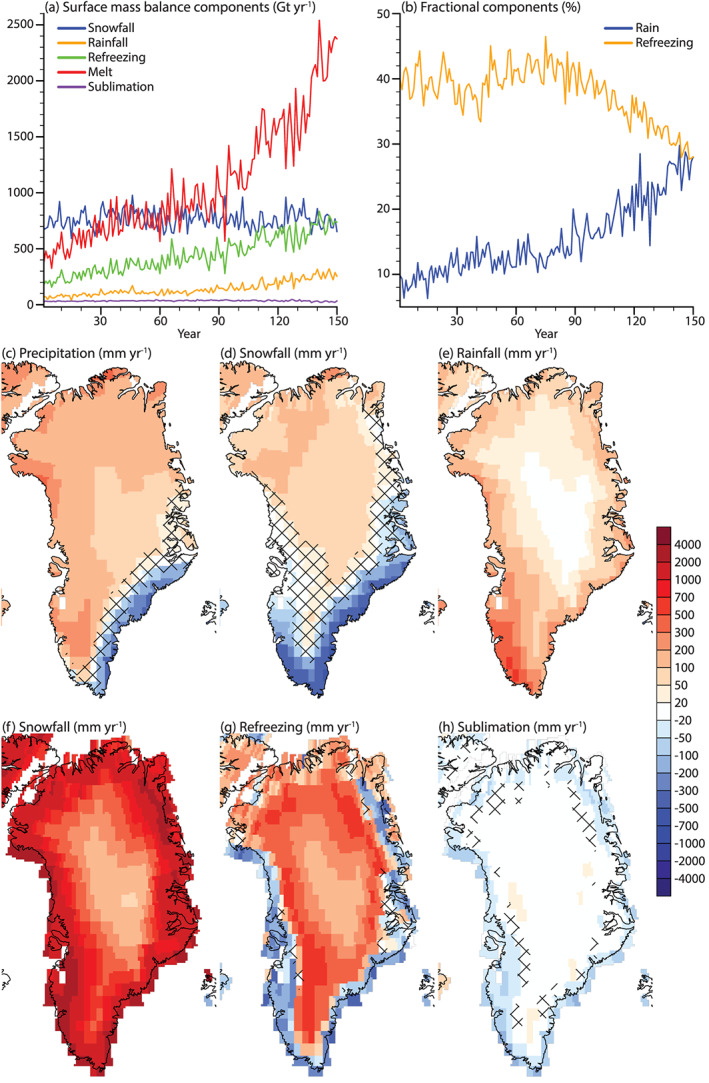
Annual ice sheet integrated (a) SMB components (Gt yr^−1^) and (b) fractional components (%). In (a) we show snowfall (blue), rainfall (yellow), refreezing (green), melt (red), and sublimation (purple). In (b) we show rain fraction (blue; defined as the fraction of rain to total precipitation) and refreezing fraction (yellow; the ratio of refreezing to melt and rainfall). Annual anomaly maps of the last 20 years of 1PCT compared to CTRL for selected SMB components (mm yr^−1^). (c) Precipitation, (d) snowfall, (e) rainfall, (f) surface melt, (g) refreezing, and (h) sublimation. Note the nonlinear color scale. Only values corresponding to the glaciated part of the grid cell are shown. Areas showing non‐significant changes are patched.

To understand the processes contributing to the large and rapid decline in SMB, particularly after year 90, we investigate individual SMB components. Precipitation increases over most parts of the ice sheet (Figure  5c). The highest increases are in the high accumulation area in the North‐West, in the South‐West, and at the northern margin. Precipitation decreases along the high accumulation area in the South‐East, likely due to reduced cyclogenesis in the Greenland sea.

Snowfall, the largest SMB component in the CTRL simulation (Table 1), shows a non‐significant decrease of −6 ± 78 Gt yr^−1^ (Figure 5a and Table 1). This decrease is likely related to (multi‐)decadal variations in the snowfall. This result is in contrast to other studies (Fettweis, Franco, et al., 2013; Mottram et al., 2017; Rae et al., 2012; van Angelen et al., 2013; Vizcano et al., 2014) which show increased snowfall over the GrIS in 21st‐century projections. Although the integrated snowfall does not significantly change during the simulation, significant local changes in snowfall are apparent in Figure 5d. In the interior and the north, snowfall increases. The map of increased snowfall corresponds well to the map of where SMB increases (Figure 4e). At the South‐West margin, snowfall decreases due to higher temperatures causing the precipitation to fall as rain. At the South‐East margin, less snowfall is caused by both a higher fraction of rainfall and less total precipitation (Figure 5c).

Rainfall increases significantly with 180 ± 33 Gt yr^−1^ (Figure 5a and Table 1) by 131–150, which is a threefold increase. The time series reveals that the trend in rainfall is small (Table 1) before year 90. After year 90, the trend becomes positive. Part of the explanation of this positive trend is the general precipitation increase from a warmer and moister atmosphere. Additionally, due to the warmer atmosphere over the ice sheet, a higher fraction of precipitation falls as rain (Figure 5b). The fraction of precipitation falling as rain on the GrIS goes from 8% to 27%. Increased rainfall is robust among projections of future GrIS SMB. Spatially, rainfall increases everywhere on the ice sheet (Figure 5e). The largest increase in rainfall is in the South‐West.

Melt production at the surface of the GrIS increases significantly with 1,540 ± 317 Gt yr^−1^ (Figure 5a and Table 1), and thereby, melt becomes the largest SMB component around year 90 (Δ*T*_*global*_ = 2.7 K). After year 90, the positive melt trend increases (Table 1). Melt increases significantly over the entire ice sheet (Figure  5f). The increase in the melt is topographically dependent, with the largest increases at the margins (low elevation) and the smallest increases in the interior (high elevation).

Refreezing, the amount of available water at the surface from surface melt and rain that is being refrozen in the snow mass significantly increases with 466 ± 83 Gt yr^−1^. The rate of refreezing increase is positive and significant (Table 1) in the first 90 years of the simulation. After year 90, the refreezing increase accelerates (Table 1). The refreezing capacity (the fraction of refrozen water to available water at the surface) decreases at the start of the 1PCT simulation up to year 40 (Figure 5b), whereafter the refreezing capacity recovers for a period of ∼40–50 years. After this period, the refreezing capacity continuously declines until the end of the simulation. The reason for this latter rapid loss of refreezing capacity is that the melt generation and rainfall increases are largest in areas experiencing larger loss of snow mass. As a result, refreezing decreases in areas where the melt increase is highest (Figure 5g).

The integrated sublimation change in the 1PCT is −1 ± 7 Gt yr^−1^ (not significant). However, the anomaly map of sublimation reveals significant local changes (Figure 5h). Along the margins, the sublimation decreases, while in the accumulation area, sublimation increases. These changes can be explained through changes in LHF, which will be addressed in the next subsection.

### 4.3 SEB Evolution

**Figure 6:**
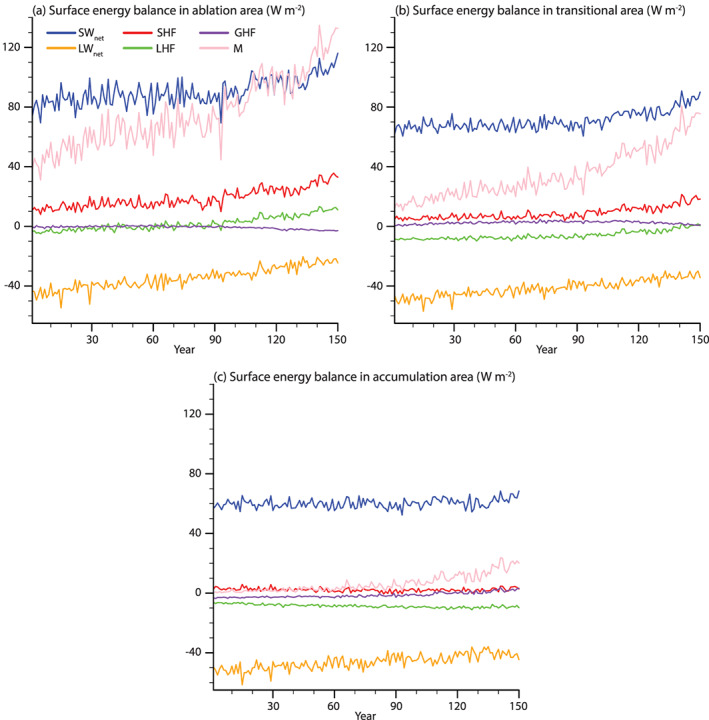
Summer (JJA) ice sheet averaged surface energy balance components (W m^−2^) in (a) ablation area, (b) transitional area, and (c) accumulation area. The ablation area is defined as the ablation area in the CTRL simulation. The transitional area is defined as the area that is accumulation area in the CTRL simulation but transition into ablation area during the 1PCT. The accumulation area is the area of the ice sheet that is an accumulation area in the CTRL and remains an accumulation area through the 1PCT simulation. The components shown are SW_*net*_ (blue), LW_*net*_ (yellow), SHF (red), LHF (green), GHF (purple), and melt energy (pink) (all in units of W m^−2^. Positive values mean increased energy at the ice sheet surface.

**Figure 7:**
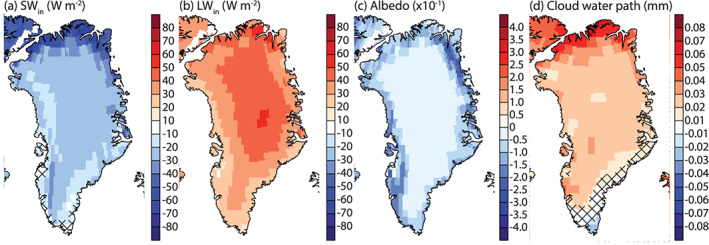
Summer (JJA) anomaly maps of the last 20 years of 1PCT compared to CTRL for selected radiation influent quantities. (a) Incoming solar radiation (W m^−2^), (b) incoming longwave radiation (W m^−2^), (c) albedo (‐), and (d) cloud water path (mm). Only values corresponding to the glaciated part of the grid cell are shown. Areas showing no significant change are patched.

**Figure 8:**
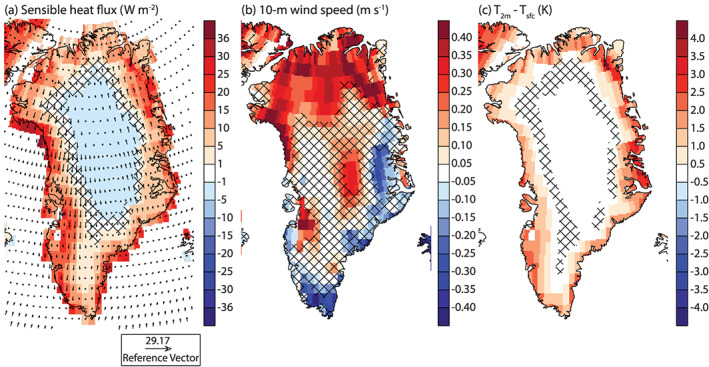
Summer (JJA) anomaly maps of the last 20 years of 1PCT compared to CTRL for selected sensible heat flux influential quantities. (a) Sensible heat flux (W m^−2^), (b) 10‐m wind speed (m s^−1^), and (c) *T*_2*m*_‐*T*_*sfc*_ (K). Note the nonlinear color scale for (a). Only values corresponding to the glaciated part of the grid cell are shown. Arrows on (a) indicate wind speed and direction at 850 hPa. Areas showing no significant change are patched.

Figure 6 shows the evolution of summer SEB components. With the exception of GHF, all SEB components significantly increase in the ablation area (Figure 6 and Table 2). This results in a 69.8 ± 12.4 W m^−2^ increase in melt energy by 131–150. Until year 100, SW_*net*_ only increases slightly and after that stabilizes. This is due to the compensation of a decreased SW_*in*_ due to thicker clouds and a decreased albedo (Figures 7a and 7c and Table 2). After year 100, SW_*net*_ increases as the SW_*in*_ stabilizes while the albedo continues to decrease. LW_*net*_ is the largest contributor to the melt energy increase (Table 2). This is caused by increased LW_*in*_ (Figure  7b and Table 2), caused by more emission of longwave radiation from the atmosphere to the surface as the atmosphere warms. SHF increase (Table 2) is caused by atmospheric warming, more heat advected over the ice sheet, and the difference between *T*_2*m*_ and *T*_*sfc*_ becoming larger (Figure 8) as the ice sheet surface has an upper limit of warming to 0°C. LHF increases (Table 2). There is a regime shift around year 80, where LHF goes from being negative during the summer to positive, likely due to the higher amount of moisture held by the atmosphere together with lengthened bare ice exposure. GHF decreases as the refreezing is much lower in the ablation area (Table 2).

**Table 2:** Surface Energy Balance Components in the CTRL, the Last 20 Years of 1PCT, and Their Difference for the Ablation Area, Transitional Area, and the Accumulation Area

Component	CTRL (W m^−2^)	1PCT (W m^−2^)	Difference (W m^−2^)
*Ablation area*
SW_*in*_	251.7 ± 6.9	226.1 ± 8.0	− **25.6**
SW_*net*_	82.7 ± 5.8	102.7 ± 6.9	**20.2**
LW_*in*_	260.2 ± 3.1	289.4 ± 2.4	**29.2**
LW_*net*_	−44.3 ± 2.7	−23.9 ± 2.0	**20.4**
SHF	11.8 ± 2.1	29.7 ± 4.0	**17.9**
LHF	−3.6 ± 1.2	10.0 ± 2.0	**13.6**
GHF	−0.2 ± 0.5	−2.5 ± 0.4	− **2.3**
Melt energy	46.4 ± 7.1	116.2 ± 12.4	**69.8**
*Transitional area*
SW_*in*_	279.6 ± 4.6	253.0 ± 5.2	− **26.5**
SW_*net*_	66.5 ± 2.7	82.8 ± 5.4	**16.3**
LW_*in*_	245.6 ± 3.4	277.5 ± 2.7	**31.9**
LW_*net*_	−48.0 ± 2.5	−32.7 ± 2.0	**15.4**
SHF	5.1 ± 1.0	16.2 ± 3.1	**11.2**
LHF	−8.7 ± 0.6	−0.4 ± 1.8	**8.2**
GHF	1.5 ± 0.6	1.4 ± 0.6	−0.1
Melt energy	16.4 ± 2.7	67.4 ± 9.3	**51.0**
*Accumulation area*
SW_*in*_	300.1 ± 4.6	276.6 ± 4.3	− **23.5**
SW_*net*_	59.5 ± 2.2	62.5 ± 3.5	**3.0**
LW_*in*_	220.0 ± 4.9	260.7 ± 3.4	**40.7**
LW_*net*_	−51.7 ± 2.8	−40.4 ± 2.5	**11.2**
SHF	2.9 ± 1.0	3.0 ± 1.4	0.1
LHF	−6.8 ± 0.5	−0.4 ± 1.8	− **2.3**
GHF	−2.9 ± 0.3	1.7 ± 1.1	**4.6**
Melt energy	1.0 ± 0.5	17.6 ± 4.3	**16.6**

*Note*. Differences in bold are significant. The ± indicates the 1 standard deviation.

In the transitional area, the melt energy increases with 51.0 ± 9.3 W m^−2^ (Figure 6b and Table 2), somewhat less than in the ablation area. The largest contributor to this increase is the SW_*net*_. Also here, the compensation between SW_*in*_ and albedo maintains a stable SW_*net*_ in the first decades. Albedo decrease accelerates after year 90 as snow mass decreases, and there is more bare ice exposure, leading to increased SW_*net*_. In the last 30 years, SW_*in*_ does not further decrease. LW_*net*_ increases (Table 2) for the same reason as in the ablation area. However, the increase in LW_*net*_ is less, due to the compensation of increasing LW_*out*_ as the surface temperature increases. As in the ablation area, SHF is stable in the first 90 years, whereafter it increases for the same reasons. However, the increase is less than in the ablation area (Table 2), likely due to the *T*_*sfc*_ being at the melting point for a shorter period than in the ablation area during the summer. The same mechanism leading to increased LHF in the ablation area leads to an LHF increase in the transitional area. With this increase, the 20‐year summer mean indicates this area has not transitioned from being dominated by sublimation to condensation. GHF shows no significant change, likely due to a competition between increased water available for refreezing and reduced refreezing capacity.

Also, the melt energy increases in the accumulation area (16.6 ± 4.3 W m^−2^, Table 2, and Figure 6c) by 131–150. The SW_*net*_ only increases slightly in the last 30 years of the simulation, for the same reasons as the SW_*net*_ showing a late response in the other areas. As the albedo change in this area is rather small (Figure  7c), the SW_*net*_ increase is also smaller than in the other areas. LW_*net*_ is the largest contributor to increased melt energy for the accumulation area (Table 2). This increase is caused by enhanced LW_*in*_ due to increased cloud thickness and higher atmospheric temperatures. SHF shows no significant change, due to a very small change in the difference between *T*_2*m*_ and *T*_*sfc*_. On the other hand, the LHF decreases and becomes more negative, indicating more energy is used for sublimation. GHF increases due to more melting in the accumulation area, allowing for more refreezing, which releases heat in the snowpack.

Figure 7 shows spatial maps of SW_*in*_, LW_*in*_, albedo, and cloud water path (CWP) anomalies. SW_*in*_ decreases the most in the north due to increased cloud fraction (Figure 3b) and increased CWP (Figure 7d). Over large parts of the ice sheet, the decrease in SW_*in*_ is between 20 and 30 W m^−2^. This smaller change is due to no change in cloud fraction and a smaller increase in cloud thickness. Increases in LW_*in*_ show a different pattern than decreases in SW_*in*_. Except for the north, the LW_*in*_ increase shows a topographically and latitudinal dependent pattern. The topographically dependent pattern is caused by summer atmospheric warming being stronger at higher elevations. A positive south‐to‐north gradient causes the latitudinal pattern in cloud fraction and thickness change. Albedo decreases significantly over the entire ice sheet (Figure  7c). The largest increases are found along the margins, particularly in regions covered permanently by snow now has bare ice exposure. Also, we expect in areas with seasonal snow cover at the margins, that the bare ice exposure is prolonged.

We showed that the melt energy accelerates after year 90, causing larger amounts of surface melt and results in an accelerated SMB decrease. SHF contributes to this acceleration over the regions of the GrIS, producing the largest amounts of melt. The SHF increases the most at the margins (Figure 8a). In the interior, the SHF slightly decreases. The 850‐hPa winds over Greenland are cyclonic, which is the pattern associated with a positive phase of NAO.

The 10‐m wind speed (Figure 8b), a proxy for the strength of turbulent transfer between atmosphere and surface, only changes significantly in the north, at the summit, locally in the southwest and along the southeastern margin. Over large parts of the northern ice sheet, wind speeds increase. Also, in the west, wind speeds increase locally. On the other hand, in the South‐East, wind speed decreases of up to the same magnitude appear.

The temperature difference between the near‐surface atmosphere and the surface increases the most in the ablation and transitional area. In this area, the surface reaches the melting point and cannot further increase its temperature while the *T*_2*m*_ continues to increase in response to the CO_2_ forcing. This increase in the difference between *T*_2*m*_ and *T*_*sfc*_ increases the SHF.

## 5 Effects of North Atlantic Atmospheric Circulation Change on GrIS SMB

**Figure 9:**
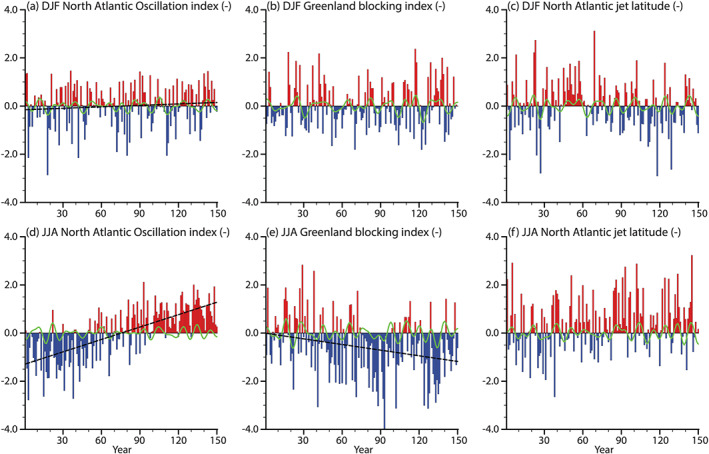
Indices of North Atlantic circulation metrics in red (positive) and blue (negative) bars. The upper row shows DJF means, and the lower row shows JJA means of (a, d) North Atlantic Oscillation, (b, e) Greenland blocking index, and (c, f) jet latitude. All indices are standardized with respect to CTRL. Significant trends are indicated with a dashed black line. The green line shows 10‐ to 30‐year band‐pass filtered time series.

The aim of this section is to (1) explore variability and trends in North Atlantic circulation and (2) investigate its potential impact on GrIS precipitation and melt. For the first part, we examine the evolution of the indices for the NAO, GBI, and latitudinal position of the North Atlantic jet, with the metrics outlined in section 2.3.2 and separately for winter and summer (Figure 9).

The winter NAO exhibits a small, but a significant trend toward its positive phase during years 1 to 150 of the 1PCT simulation. On the contrary, the winter GBI index does not exhibit a significant trend. As the winter GBI, the winter jet latitudinal position does not have a significant trend.

The summer NAO exhibits a strong significant trend toward its positive phase in response to CO_2_ forcing. As seen in Figure 8a, we do see a circulation anomaly related to this phase of the NAO. The GBI exhibits a significant negative trend toward its negative phase. We find that these two indices are correlated to SSTs around southern Greenland (Figure S3), which might be causing the trends in these two indices. The jet stream, on the other hand, does not significantly change its position.

**Figure 10:**
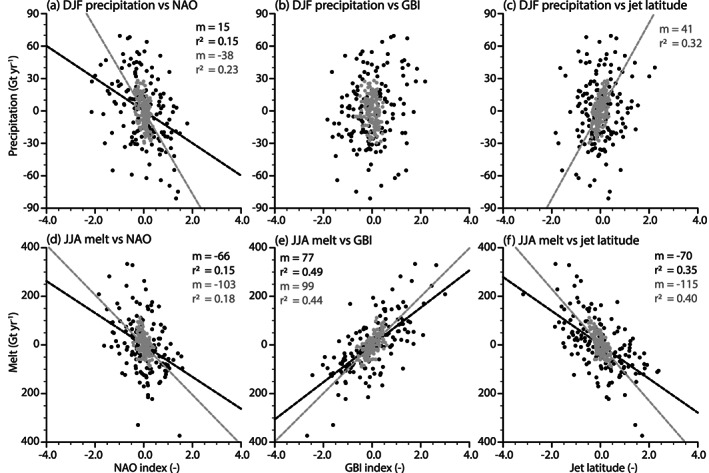
DJF GrIS‐integrated filtered precipitation (Gt yr^−1^) regressed onto DJF North Atlantic circulation filtered indices (upper row) and JJA GrIS‐integrated filtered surface melt (Gt yr^−1^) regressed onto JJA North Atlantic circulation filtered indices (lower row). Circulation indices used are (a, d) NAO, (b, e) GBI, and (c, f) jet latitude. Black dots represent 10‐year high‐pass filtered quantities, and gray dots represent 10‐ to 30‐year band‐pass filtered quantities. The timescale of the filtered quantities effectively removes both the mean and the trend of each time series. Black (gray) lines are drawn where the regression is significant, with an annotated *m* (slope), and *r*^2^ for the explained variance.

Figure 10 shows linear regressions between GrIS‐integrated, summer melt, and winter precipitation and the corresponding seasonal NAO, GBI, and jet latitude indices.

The NAO and the jet position modulate the amount of winter precipitation falling on the GrIS (Figures 10a and 10c). A more positive NAO results in less precipitation. This relationship is robust through the timescales investigated here, where sub‐decadal variations in NAO explain 15% of the precipitation variability, while decadal oscillations explain 23% of the variability. For the jet stream, only its decadal component seems to have an impact on GrIS precipitation variability. On this timescale, a more northern displaced jet stream results in higher precipitation rates over the GrIS. Changes in the jet stream position account for 32% of the precipitation variability. Variations in GBI do not show any significant relationship to GrIS precipitation (Figure 10b).

The relationship between melt and NAO is significant, on both sub‐decadal and decadal timescales. A more negative NAO implies higher melt rates. The change in the melt due to decadal variations in the NAO is stronger than on sub‐decadal timescales. Sub‐decadal variations in the NAO explain 15% of melt variability, while 18% is explained for decadal variations in melt variability. A relationship between GBI and surface melt is also shown (Figure 10e). On sub‐decadal timescales, the GBI explains 49% of the surface melt variability. The GBI also shows to have a strong influence on surface melt on decadal timescales, explaining around 44% of the variation. The NAO and the GBI trends toward its positive and negative phases, respectively (Figure  9d), while the melt increases in response to higher CO_2_ (Figure 5). This result shows that the NAO and GBI are not the main drivers of increased melt. Instead, the relationship suggests that the change in NAO and GBI counteracts the effect of global warming on the surface melt. Our simulation also shows a relationship between the position of the jet stream and the GrIS surface melt (Figure 10f). A more southern displaced jet stream is related to more surface melt, on both sub‐decadal and decadal timescales. In contrast to the GBI, a change in jet position explains more of surface melt variability on decadal timescales (40%) than on sub‐decadal timescales (35%).

## 6 Summary and Discussion

This study projects the transient climate and GrIS SMB response to increasing CO_2_ forcing until quadrupled pre‐industrial levels with a fully coupled Earth System Model. With respect to previous work that focuses on regional climate modeling or simplified melt calculations from global models, the main novelty is in the detailed analysis of GrIS surface energy and mass budgets using a global climate model coupled with a realistic representation of ice sheet snow and firn processes (van Kampenhout et al., 2017).

CESM2.1 simulates a global mean temperature anomaly of 5.3 ± 0.4 K by the end of the simulation (years 131–150). The model has a high equilibrium climate sensitivity of 5.3 K (Gettelman et al., 2019) to CO_2_ forcing compared to models from the CMIP5 (Taylor et al., 2012) that simulate equilibrium climate sensitivities in the range of 2.1–4.7 K (Andrews et al., 2012). At the same time, CESM2.1 projects high reductions in NAMOC (Figure S1). Both high climate sensitivity and NAMOC reduction are important controls on the GrIS SMB response to CO_2_ forcing.

The simulated Arctic amplification (ratio between mean temperature >60°N and global mean temperature) is 1.6 (years 131–150). Major summer contributions to Arctic warming are loss of snow over the terrestrial Arctic, associated albedo feedback, and decreased cloud cover. In winter, the main contribution is from sea ice loss. The Arctic amplification factor was found to be 1.5–4.5 in CMIP3 (Holland & Bitz, 2003). Also, a previous study with CESM version 1.0 found an amplification factor of 2.1 (Vizcano et al., 2014). So the Arctic warming, compared to the global warming found here, is in the lower range. This may be due to the Arctic sea ice being biased thin in CESM2.1. Further, we find that the Arctic becomes seasonally sea ice free at a global warming of 2.1 K. A September sea ice‐free Arctic is a robust response (Snape & Forster, 2014) to representative concentration pathway (RCP) 8.5 forcing, which ends with a CO_2_ forcing of >1,370 ppm (which is similar to the final CO_2_ of 1,140 ppm in this study). The timing of seasonal sea ice‐free conditions under RCP8.5 forcing is estimated as ∼2,040–2,060 in Wang and Overland (2012) and Snape and Forster (2014).

The SMB of the GrIS decreases with 994 Gt yr^−1^ in our simulation. In a similar CESM2.1 study, though with a dynamically evolving ice sheet, the SMB decreases with 952 Gt yr^−1^ by the same time (Muntjewerf et al., 2020). This SMB decrease represents a 2.8 mm yr^−1^ contribution to global mean sea‐level rise, assuming that the pre‐industrial ice sheet SMB would give no change in sea‐level rise. Compared to Church et al. (2013), this contribution is at the high end, likely due to the high climate sensitivity simulated here. The main contributor of the SMB decrease is a melt increase of 1,540 Gt yr^−1^. This melt is higher than what is projected under an RCP8.5 scenario (600–700 Gt yr^−1^;Fettweis, Franco, et al., 2013; Rae et al., 2012; Vizcano et al., 2014), likely due to the higher climate sensitivity. The refreezing is here projected to increase with 466 Gt yr^−1^, which is a factor of 0.30 to the melt increase. Rae et al. (2012) find this factor to be in the range 0.19–0.45, depending on the forcing model and RCM. Vizcano et al. (2014)) find this factor to be 0.21, likely in connection with limited refreezing capacity from an absence of firn modeling as GrIS snow thickness is capped at 1 m of water equivalent.

An interesting feature of the SMB decrease is the pause in the decrease around the years 120–135 (Figure  4a). Figure 5a shows that this timing corresponds to a time of temporarily increased snowfall, and a pause in surface melt increase. The reason for this pause in surface melt increase is due to a pause in the increase of SHF and SW_*net*_ in the ablation area (Figure 6a). Due to the low summer GBI together with high summer NAO in these years (Figures 9d and 9e), it is likely that reduction in warm air advection compensates for increased atmospheric warming and temporarily prevents further albedo‐melt feedback.

Increases in GrIS precipitation are projected in state‐of‐the‐art studies (Fettweis, Franco, et al., 2013;Mottram et al., 2017; Vizcano et al., 2014). These projected increases in both snow and rainfall, while CESM2.1 projects only rainfall increase of 180 ± 33 Gt yr^−1^. Further, the spatial patterns of precipitation change modeled here differ substantially from other studies (e.g., Mottram et al., 2017) that find the maximum increase in the area where CESM2.1 projects decreased precipitation. In our simulation, this decrease is attributed to regional cooling from NAMOC reduction, which induces stabilization of the atmosphere in the North Atlantic and the Greenland sea and reduces storminess in South‐East Greenland.

At a global mean surface temperature increase of 2.7 K, we find that SMB decrease accelerates (from −2.5 ± 0.4 Gt yr^−2^ to −15.9 ± 1.1 Gt yr^−2^). This threshold temperature may be subject to change if we considered an evolving ice sheet. Gregory et al. (2004) found this temperature to be a threshold for GrIS deglaciation, as melt becomes larger than snowfall, which is in line with our findings. The surface mass loss acceleration is due to melt acceleration, together with loss of refreezing capacity. The latter has previously been identified as a key driver to the accelerated ice sheet and ice caps surface mass loss (Noël et al., 2017; van Angelen et al., 2013). The main contributor to melt increase before acceleration is the LW_*net*_. The summer LW_*net*_ increases due to higher atmospheric temperatures and thicker clouds over the GrIS in this season. At the time of acceleration, a large fraction of the GrIS reaches the melting point during the summer. This accelerates the SHF over the ablation areas, as the difference between the surface and air temperatures increases at the rate of the air temperature increase. This further contributes to an expansion of the ablation area, which exposes more bare ice leading to accelerated solar radiation absorption from the albedofeedback.

The simulated ablation area expansion emerges from background variability already at a global mean temperature increase of 1.0 K with respect to pre‐industrial, decades before SMB (decrease) emergence. Therefore, we suggest that the monitoring of ablation area expansion can be used as a precursor for the detection of an emerging anthropogenic signal in SMB.

The most SMB‐relevant changes in North Atlantic atmospheric circulation projected by CESM2.1 are trends toward more positive NAO in the winter and summer and more negative GBI in summer. High GBI in recent decades has been proposed to explain a large fraction of the recent melt increase (Delhasse et al., 2018; Hanna et al., 2018). The increase in the NAO index is robust among the CMIP5 ensemble (Gillett & Fyfe, 2013). In winter, we find that a positive NAO is related to less precipitation over the GrIS. The simulated summer trends in both NAO and GBI indices are apparent in the CMIP5 models (Hanna et al., 2018) and cause partial reduction of melt. From this, we suggest that care must be taken when extrapolating the current circulation anomaly to the future, as, for example, potential NAMOC weakening may result in future atmospheric circulation changes that reduce melt. On the other hand, there is no guarantee that the models have a correct representation of, for example, future NAMOC weakening and the current observed anomalous circulation pattern may continue to intensify.

## Supporting information



Figure S1

Figure S2

Figure S3

## Data Availability

Computing and data storage resources, including the Cheyenne supercomputer (https://doi.org/10.5065/D6RX99HX), were provided by the Computational and Information Systems Laboratory (CISL) at the National Center for Atmospheric Research (NCAR). The material is based upon work supported by NCAR, which is a major facility sponsored by the National Science Foundation under cooperative agreement no. 1852977. The CESM project is supported primarily by the National Science Foundation.
