# Supplementary figures and images for: Global Warming Threshold and Mechanisms for Accelerated Greenland Ice Sheet Surface Mass Loss
Source: J Adv Model Earth Syst. 2020 Sep 9;12(9):e2019MS002029. doi: 10.1029/2019MS002029 (PMC7540049; doi:10.1029/2019MS002029)

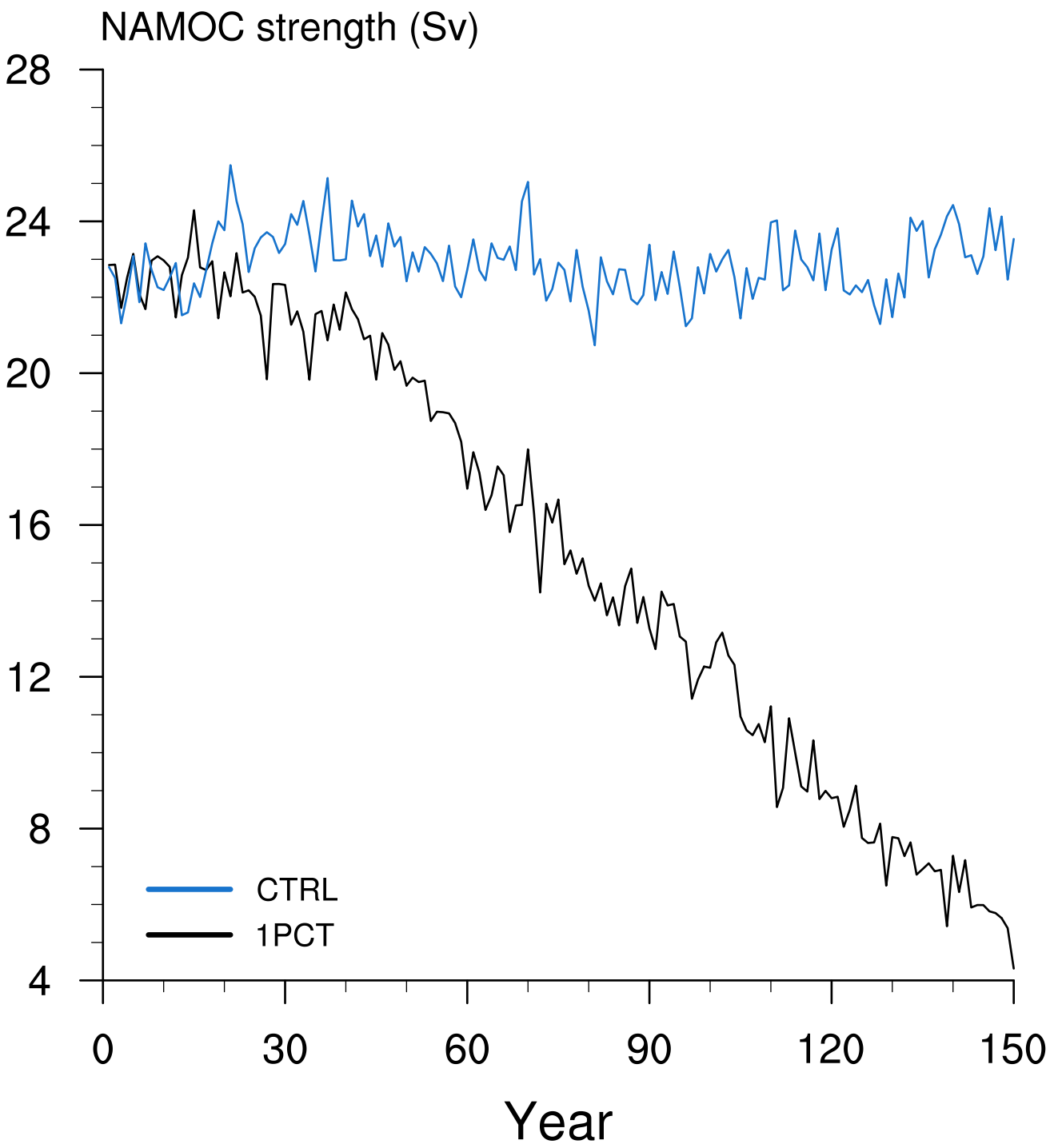

Supplement: Supplementary file 1 — Figure S1 [file JAME-12-e2019MS002029-s001.pdf]

Storm track anomaly (m)

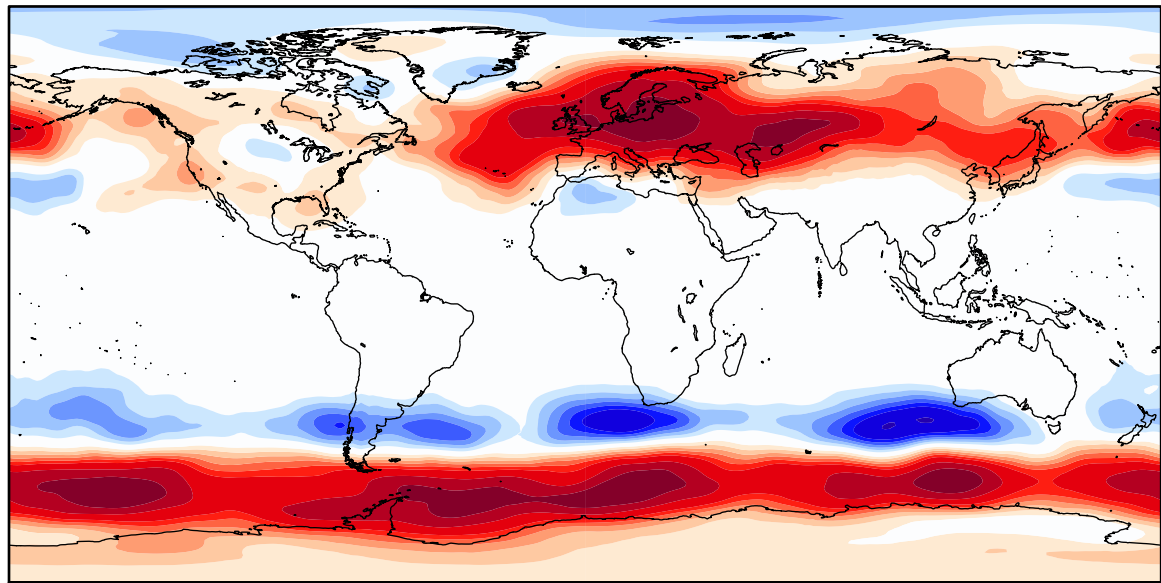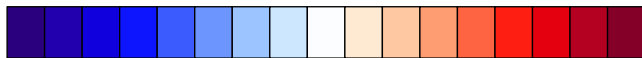

-5 -4 -3 -2.5 -2 -1.5 -1 -0.5 0.5 1 1.5 2 2.5 3 4 5

Supplement: Supplementary file 2 — Figure S2 [file JAME-12-e2019MS002029-s003.pdf]
