# Supplementary material for: Global Warming Threshold and Mechanisms for Accelerated Greenland Ice Sheet Surface Mass Loss
Source: J Adv Model Earth Syst. 2020 Sep 9;12(9):e2019MS002029. doi: 10.1029/2019MS002029 (PMC7540049; doi:10.1029/2019MS002029)
Supplement: Supplementary file 3 — Figure S3 [file JAME-12-e2019MS002029-s002.pdf]

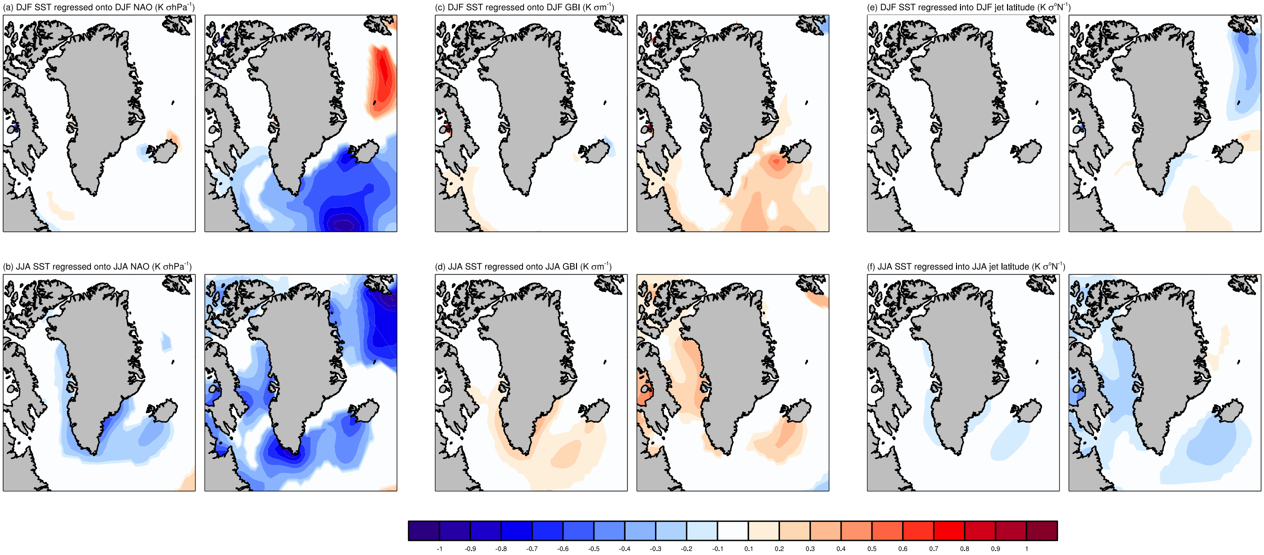

(a) DJF SST regressed onto DJF NAO (K σhPa-1)
(c) DJF SST regressed onto DJF GBI (K σm-1)
(e) DJF SST regressed into DJF jet latitude (K σ°N-1)
(b) JJA SST regressed onto JJA NAO (K σhPa-1)
(d) JJA SST regressed onto JJA GBI (K σm-1)
(f) JJA SST regressed into JJA jet latitude (K σ°N-1)
-1 -0.9 -0.8 -0.7 -0.6 -0.5 -0.4 -0.3 -0.2 -0.1 0.1 0.2 0.3 0.4 0.5 0.6 0.7 0.8 0.9 1
